# Genome Sequence of Mycobacterium Phage CrystalP

**DOI:** 10.1128/genomeA.00542-17

**Published:** 2017-07-13

**Authors:** Christine L. Fleischacker, Miriam Segura-Totten, SEA-PHAGES 2016 Bioinformatics Workshop, Rebecca A. Garlena, Deborah Jacobs-Sera, Welkin H. Pope, Daniel A. Russell, Graham F. Hatfull

**Affiliations:** a Department of Biology, University of Mary, Bismarck, North Dakota, USA; b Department of Biology, University of North Georgia, Dahlonega, Georgia, USA; c Department of Biological Sciences, University of Pittsburgh, Pittsburgh, Pennsylvania, USA; d HHMI Science Education Alliance-Phage Hunters Advancing Genomics and Evolutionary Science Program, Chevy Chase, Maryland, USA

## Abstract

Mycobacteriophage CrystalP is a newly isolated phage infecting *Mycobacterium smegmatis* strain mc^2^155. CrystalP has a 76,483-bp genome and is predicted to contain 143 protein-coding and 2 tRNA genes, including repressor and integrase genes consistent with a temperate lifestyle. CrystalP is related to the mycobacteriophages Toto and Kostya and to other Cluster E phages.

## GENOME ANNOUNCEMENT

The large collection of phages with sequenced genomes that infect the same host strain, *Mycobacterium smegmatis* mc^2^155, reveals substantial diversity and at least 30 separate lineages represented as clusters and singletons (1, 2). Mycobacteriophage CrystalP was isolated from soil collected from North Huntingdon, PA, using enrichment and *M. smegmatis* mc^2^155 as the host. CrystalP forms plaques 2 mm in diameter with a clear center and turbid halo, and examination with electron microscopy reveals that it is a member of the *Siphoviridae*, with a 70-nm-diameter capsid and a flexible, noncontractile tail that is 240 nm in length. Double-stranded DNA was isolated and sequenced using an Illumina MiSeq 150-bp single-end run. Trimmed reads were assembled using Newbler, yielding a 76,483-bp contig with 500-fold coverage, and the viral genome was determined to have 9-base 3′ single-stranded extensions (5′-CGCTTGTCA). The G+C content is 63.0%.

The CrystalP genome was annotated using DNA Master (http://cobamide2.bio.pitt.edu/), Glimmer (3), GeneMark (4), Aragorn (5), tRNAscan-SE (6), BLASTP (7), HHPred (8), and Phamerator (9). Annotation identified 145 protein-coding genes, 43 of which were assigned putative functions, and 2 tRNA genes. CrystalP is a member of Cluster E, one of the least diverse mycobacteriophage clusters, and its closest relative is mycobacteriophage Toto (GenBank accession number JN006061), with which it shares 99% nucleotide identity spanning 99% of their genome lengths.

The CrystalP genome is organized with its virion structure and assembly genes in the leftmost 30 kbp of the centrally located lysis and immunity functions and its nonstructural genes in the rightmost 35 kbp of the genome. Most of the genes are transcribed rightward, with the exceptions of a group of 14 small (<500 bp) leftward-transcribed genes centrally located between the lysis and immunity functions and a set of 16 leftward-transcribed genes near the right genome end; the putative immunity repressor (gene *55*) is also transcribed leftward. CrystalP encodes a putative recombination system with an exonuclease (gene *66*) and Erf-like DNA pairing proteins, in addition to a RecA-like protein (gene *116*). It also codes for an RNA ligase (gene *90*) and a polynucleotide kinase (gene *87*) implicated in tRNA repair, along with 2 tRNAs (tRNA^arg^ and tRNA^gly^, genes *107* and *108*, respectively), which collectively may play roles in countering host defenses (10). CrystalP also encodes an adenine-specific DNA methyltransferase (gene *98*), which is present in most but not all Cluster E genomes as well as in some Cluster F genomes, and which may play a role in defending against host restriction systems.

Approximately 57% of the CrystalP-predicted protein-coding genes are not present outside the group of 84 Cluster E phages; these include many of the putative virion structure and assembly genes (terminase large subunit, portal, protease, capsid, head-to-tail connectors, major tail subunit, tape measure protein, and two minor tail subunit genes). We noted that the putative immunity repressor (gene *55*) is conserved among all 84 of the Cluster E phages and these may form a homoimmune group.

### Accession number(s).

CrystalP is available at GenBank with accession number KY319168.
